# Supplementary material for: An evaluation of interventions within a Growth Through Nutrition project aimed at enhancing optimal nutrition and water, sanitation and hygiene (WASH) and nutrition practices among nutritionally most vulnerable households (MVHHs) in Ethiopia
Source: PLoS One. 2024 Oct 31;19(10):e0309426. doi: 10.1371/journal.pone.0309426 (PMC11527315; doi:10.1371/journal.pone.0309426)
Supplement: Attachment — Submitted filename: Response to Reviewers.docx [file pone.0309426.s002.docx]

**Dear Dr. Muhammad,**

Your valuable comments and suggestions are sincerely appreciated. We have reviewed and responded to each comment provided by the editor and reviewers, as detailed below.

Best regards,

Cherinet

**Journal requirements:**

If applicable, we recommend that you deposit your laboratory protocols in protocols. …

**Author’s response**: Not applicable

1. Please ensure that your manuscript meets PLOS ONE's style requirements, including those for file naming. The PLOS ONE style templates can be found at

<https://journals.plos.org/plosone/s/file?id=wjVg/PLOSOne_formatting_sample_main_body.pdf> ...

a. **Author’s response:** The manuscript was prepared following PLOS ONE's style requirements. The files are named as "Revised Manuscript" with track changes and "Manuscript" without track changes.

2. We note that the grant information you provided in the ‘Funding Information’ and ‘Financial Disclosure’ sections do not match.
   a. **Authors Response:** The correct grant number is “USAID Cooperative Agreement No. AID 663-A-16-00007.” The authors used this funding to conduct the study.
3. If there are ethical or legal restrictions on sharing a de-identified data set, please explain them in detail …
   a. **Author’s response:** There are no specific ethical/legal restrictions in accessing the dataset. However dataset will be made public by the funding agency, via USAID Development Data Library (DDL), https://data.usaid.gov/browse?category=Health, to which we have submitted all raw data
4. Your ethics statement should only appear in the Methods section of your manuscript. If your ethics statement is written in any section besides the Methods, …
   a. **Author’s response:** Ethics statement is written only in methodology - in the manuscript.

**Additional editor Comments:**

1. Your title explicitly suggests that your paper discusses WASH and nutrition, however, your abstract only mentions hand washing briefly. I expected more results related to Water, Sanitation and Hygiene …
   a. **Author’s response:** The abstract now incorporates concise findings on Water, Sanitation, and Hygiene in addition to the reported hand washing practice. The title has been adjusted to better align with the study's objective. Besides, the headings on pages 10 and 12 have been condensed for clarity.
2. Objective of the study on page 4 is unclear because it has two "ands". Please rewrite it for clarity.
   a. **Author’s response**: Corrected
3. Introduction of the paper is too short. …
   a. **Author’s response**: This comment is addressed in the introduction section by inserting additional evidence meant to illustrate why access to WASH (Water, Sanitation, and Hygiene) is necessary.
4. Table 1 suggests that the sample used in this study mostly consists of ever-married women. However, this specification of the sample is not mentioned …
   a. **Author’s response**: The Study title refers to ‘Households” not specifically to ‘women and children as, the sampling is not limited to women and children but caregivers in the households who can respond for the questionnaires. The data included male (e.g. 6.6% in 2020) as shown in this table.
5. In Table 4, feeding practices are mostly decreasing between 2017 and 2018 substantially and then increasing between 2018 and 2020 again. These indicators are usually not so volatile. …
   a. **Author’s response**: This was explained in the discussion section, highlighting the necessity for additional exploration to determine the events that occurred in the study areas during this timeframe
6. What is the difference between heading on Page 10 and Page 12? children aged 6 -23 months are also under 2 years old. And in this paper, nutrition seems to be used synonymous with feeding. if that is the case, both headings are identical.
   a. **Author’s response**: Shifted the order of the sections with titles modified so that the narrative flows from ‘breastfeeding’ to ‘complementary feeding’/ Presented in two separate sections for the sake of clarity/ for better flow.
7. Please rethink the arrangement of arguments. it is confusing right now. Group similar topics together and attempt to establish a transition and relationship among the arguments. …
   a. **Author’s response:** rearranged the presentation in the Discussion section by starting with nutrition/feeding related issues among mothers and children and proceeding to WASH. This is in line with the revised order in the Results section - that goes from pregnancy, breastfeeding, complementary feeding, maternal nutrition to WASH practices. Similar rearrangement is done for the recommendation section. While headings clearly reflect the flow in the Results section, separate sections are not used in the Discussion and Recommendation sections.
8. In the discussion, talk about the objectives of the project and one by one explain what it managed to achieve and what it did not. Otherwise arguments are lost in words.
   a. **Author’s response**: Addressed together with the above comment by adding/modifying the objectives that the project aimed to fulfil and reporting related changes at each paragraph in the Discussion section.

**Reviewers' comments:**

**Reviewer's Responses to Questions:**

**Reviewer's Responses to Questions:**

1. Is the manuscript technically sound, and do the data support the conclusions? …

Reviewer #1: Yes

Reviewer #2: Yes

**Author’s response**: Ok

2. Has the statistical analysis been performed appropriately and rigorously?

**Reviewer #1**: No,

**Author’s response to reviewer #1**: We believe that the statistical analysis has been conducted properly and carefully, aligning with the objectives of the study. The objective was to evaluate changes associated with interventions aimed at Most Vulnerable Households within the Growth through Nutrition Project. Given that this is the primary aim of the study, we presume that the statistical analysis has been conducted appropriately and rigorously.

3. Have the authors made all data underlying the findings in their manuscript fully available?

**Author’s response to reviewer #1**: Raw data will be made available to public via <https://data.usaid.gov/browse?category=Health>

4. Is the manuscript presented in an intelligible fashion and written in standard English?

**Reviewer #1**: Yes

**Reviewer #2**: Yes

**Author’s response**: Ok

5. Review Comments to the Author

**Reviewer #1**: This is a good study, however, the analysis could have included other dimensions without involving advanced econometric techniques. For instance, it would be interesting to explore how WASH practices and nutritional intake of the infants change with education of the mother. The authors are suggested to add two-way and three-way cross tabulations.

**Author’s response**: This is a good recommendation. However, as the primary objective of the study was limited to assessing changes over time among project supported households and changes associated with project-specific intervention (e.g. behavior change sessions) than seeking for association/correlation between other possible underlying factors.

**Reviewer #2**: Excellent study. But please add a section of behavior change models as adoption of WASH practices is a long run behaviour change. For instance, see Ginja, S., Gallagher, S., & Keenan, M. (2021). Water, sanitation and hygiene (WASH) behaviour change research: why an analysis of contingencies of reinforcement is needed. International Journal of Environmental Health Research, 31(6), 715-728.

**Author’s response**: Enriching feedback.

Our reservation is as SBCC sessions complement material support and technical assistance to MVHHs, a focus on BCC model might shift the analysis of the study more to the BCC component. As this study is not originally planned with the explanatory framework presented in the article- analysis of contingencies of reinforcement- in mind, this makes discussion based on such concept less robust.
