# Supplementary material for: An evaluation of interventions within a Growth Through Nutrition project aimed at enhancing optimal nutrition and water, sanitation and hygiene (WASH) and nutrition practices among nutritionally most vulnerable households (MVHHs) in Ethiopia
Source: PLoS One. 2024 Oct 31;19(10):e0309426. doi: 10.1371/journal.pone.0309426 (PMC11527315; doi:10.1371/journal.pone.0309426)
Supplement: Attachment — Submitted filename: Response to Reviewers.docx [file pone.0309426.s005.docx]

Dear Dr. Muhammad,

Thank you once again for your valuable comments. We have carefully reviewed and addressed each one in the manuscript. Below, you will find our responses and explanations of how the comments have been incorporated. The revised manuscript, with and without track changes, has also been uploaded to the system.

Best,

Cherinet

**Additional Editor Comments:** Please add the statement about community level water interventions in the paper and upload the manuscript again.

**Author’s response:** Regarding the comment on community-level water interventions, we crafted the following paragraph and included it in the introduction section of the manuscript: "Efforts to increase access to safe water were targeted at the community level, while household interventions focused on promoting hygienic practices such as handwashing and proper latrine construction and use. Although water supply schemes were included in the intervention, they were not exclusive to the population under study. Consequently, we did not analyze the households' water access."

**Journal Requirements**: Please review your reference list to ensure that it is complete and correct. If you have cited papers that have been retracted, please include the rationale for doing so in the manuscript text, or remove these references and replace them with relevant current references. Any changes to the reference list should be mentioned in the rebuttal letter that accompanies your revised manuscript. If you need to cite a retracted article, indicate the article’s retracted status in the References list and also include a citation and full reference for the retraction notice.

**Author’s response**: Due to the addition of new references and modifications to existing ones, adjustments were made to the references in sections beyond the Introduction to ensure consistency and accuracy throughout the document.

**Previous Academic Editor's Comments**

**Comment 1:** In title authors mention Growth Through Nutrition in smaller case. I think it is the title of the project that has been evaluated. The title of the projects should be mentioned with all first letters capital to ensure that the readers do not confuse it with simple text.

**Authors’ response**: The title has been adjusted to address reviewers' feedback on capitalization. While one reviewer suggested narrowing the focus to hand-washing practices, we retained the broader term "WASH" (Water, Sanitation, and Hygiene). This is because the study’s findings encompass not only hand-washing and hygiene practices but also the construction and household-level use of latrine facilities. However, the project’s efforts to increase access to safe water were targeted at the community level rather than specifically at Most Vulnerable Households (MVHHs). Consequently, the direct impact of improved water access is not analyzed here.

**Comment 2:** Please write abstract in one paragraph and attempt to make it a little bit more concise.

**Authors’ response**: The abstract has been condensed into a single, cohesive paragraph. This reorganized version briefly summarizes the study's scope, findings, and conclusions, enhancing clarity and focus for the readers.

**Comment 3:** Page 1 line 20: Two cohorts in 2017, 2018 and 2020 is confusing? On Page 7 lines 151 and 152, authors mention “both dependent and independent of the baseline sample”. It is unclear what authors mean by it. Please explain. Also, some tables have information about Cohorts in 2020, others don’t. Please make it consistent.

**Authors’ response**: This was revised in the manuscript to address the reviewer's concerns and prevent any potential confusion.

**Comment 4:** Page 2 Line 31, thank you for adding some information about water and sanitation as well. However, it seems as if the study was primarily focused on handwashing and there is not much information about water and sanitation. It is completely fine if this is the case. Authors can then avoid using the term WASH and mention handwashing instead. If water and sanitation were indeed analyzed then I suggest to define the type of water variables and sanitation variables studied and their corresponding findings.

**Authors’ response**: As it has been indicated in the title section, WASH is still kept (despite one reviewers’ suggestion to narrow title to handwashing practice) as the findings presented are not limited to handwashing/hygiene but also refer to construction of latrine facilities and household-level use of latrine facilities. However, activities of the project towards increasing access to safe water was targeted at community level, not specific to MVHHs, thus the direct contribution of increased access to water is not analyzed here.

**Comment 5:** Page 4 lines 92-94: please replace this part with a statement of what each broad section covers in the rest of the paper.

**Authors’ response**: Corrections have been made as recommended by the reviewers.

**Comment 6**: Please merge the objectives inside the introduction section instead of giving a separate heading for it.

**Authors’ response**: The study's objectives were integrated into the introduction section.

**Comment 7**: I apologize for not noticing it earlier but there is hardly any discussion on previous studies on this topic. Only first two paragraphs of introduction appear to mention a couple of them. It can be improved in two ways: 1) add a literature review section covering the previous studies on this topic, or 2) add these studies in the introduction section to develop the motivation, not only for this study but also for the project. It is quite possible that the project documents have mentioned these studies.

**Authors’ response**: The revised introduction now includes a comprehensive global literature review. As a result of adding new references and modifying existing ones, adjustments were made to the references in sections outside the Introduction to maintain consistency and accuracy throughout the document.

**Comment 8**: Please mention sources of the tables and figures at their bottom.

**Authors’ response**: The tables and figures in this manuscript are derived directly from the raw data generated during the study. They are original and have not been sourced or quoted from any external references.

**Comment 8**: In the recommendations section, I suggest that authors should avoid stating the results. If these results are not mentioned in the results section, I suggest to move them to the results section otherwise simple suggest the key recommendations in this section. It is, in my opinion, too lengthy at the moment because it is stating results.

**Authors’ response**: It is revised to limit the inclusion of findings/ results in the recommendation section, as per the reviewer’s feedback.
==================

Please submit your revised manuscript by Jul 29 2024 11:59PM. If you will need more time than this to complete your revisions, please reply to this message or contact the journal office at [plosone@plos.org](mailto:plosone@plos.org). When you're ready to submit your revision, log on to <https://www.editorialmanager.com/pone/> and select the 'Submissions Needing Revision' folder to locate your manuscript file.

Please include the following items when submitting your revised manuscript:

- A rebuttal letter that responds to each point raised by the academic editor and reviewer(s). You should upload this letter as a separate file labeled 'Response to Reviewers'.
- A marked-up copy of your manuscript that highlights changes made to the original version. You should upload this as a separate file labeled 'Revised Manuscript with Track Changes'.
- An unmarked version of your revised paper without tracked changes. You should upload this as a separate file labeled 'Manuscript'.

If you would like to make changes to your financial disclosure, please include your updated statement in your cover letter. Guidelines for resubmitting your figure files are available below the reviewer comments at the end of this letter.

If applicable, we recommend that you deposit your laboratory protocols in [protocols.io](http://protocols.io/) to enhance the reproducibility of your results. Protocols.io assigns your protocol its own identifier (DOI) so that it can be cited independently in the future. For instructions see: <https://journals.plos.org/plosone/s/submission-guidelines#loc-laboratory-protocols>. Additionally, PLOS ONE offers an option for publishing peer-reviewed Lab Protocol articles, which describe protocols hosted on [protocols.io](http://protocols.io/). Read more information on sharing protocols at <https://plos.org/protocols?utm_medium=editorial-email&utm_source=authorletters&utm_campaign=protocols>.

We look forward to receiving your revised manuscript.

Kind regards,

Muhammad Ali, PhD
Academic Editor
PLOS ONE

Journal Requirements:

Please review your reference list to ensure that it is complete and correct. If you have cited papers that have been retracted, please include the rationale for doing so in the manuscript text, or remove these references and replace them with relevant current references. Any changes to the reference list should be mentioned in the rebuttal letter that accompanies your revised manuscript. If you need to cite a retracted article, indicate the article’s retracted status in the References list and also include a citation and full reference for the retraction notice.

Additional Editor Comments:

Dear Authors,
I am grateful for your detailed responses and thorough incorporation of our comments. I believe that your paper is very close to a nice publishable shape. I request you to please incorporate the following comments in your next revision.

In title authors mention Growth Through Nutrition in smaller case. I think it is the title of the project that has been evaluated. The title of the project should be mentioned with all first letters capital to ensure that the readers do not confuse it with simple text.

Please write abstract in one paragraph and attempt to make it a little bit more concise.

Page 1 line 20: Two cohorts in 2017, 2018 and 2020 is confusing? On Page 7 lines 151 and 152, authors mention “both dependent and independent of the baseline sample”. It is unclear what authors mean by it. Please explain. Also, some tables have information about Cohorts in 2020, others don’t. Please make it consistent.

Page 2 Line 31, thank you for adding some information about water and sanitation as well. However, it seems as if the study was primarily focused on handwashing and there is not much information about water and sanitation. It is completely fine if this is the case. Authors can then avoid using the term WASH and mention handwashing instead. If water and sanitation were indeed analyzed then I suggest to define the type of water variables and sanitation variables studied and their corresponding findings. There are some findings mentioned in the text for sanitation but the paper is almost silent on water access which is the "W" of WASH.

Page 4 lines 92-94: please replace this part with a statement of what each broad section covers in the rest of the paper.

Please merge the objectives inside the introduction section instead of giving a separate heading for it.

I apologize for not noticing it earlier but there is hardly any discussion on previous studies on this topic. Only first two paragraphs of introduction appear to mention a couple of them. It can be improved in two ways: 1) add a literature review section covering the previous studies on this topic, or 2) add these studies in the introduction section to develop the motivation, not only for this study but also for the project. It is quite possible that the project documents have mentioned these studies. You can refer to them for guidance.

Please mention sources of the tables and figures at their bottom.

In the recommendations section, I suggest that authors should avoid stating the results again. If these results are not mentioned in the results section, I suggest to move them to the results section otherwise simply suggest the key recommendations in this section. It is, in my opinion, too lengthy at the moment because of the results.


Thank you

Muhammad Ali


[Note: HTML markup is below. Please do not edit.]


[NOTE: If reviewer comments were submitted as an attachment file, they will be attached to this email and accessible via the submission site. Please log into your account, locate the manuscript record, and check for the action link "View Attachments". If this link does not appear, there are no attachment files.]

While revising your submission, please upload your figure files to the Preflight Analysis and Conversion Engine (PACE) digital diagnostic tool, <https://pacev2.apexcovantage.com/>. PACE helps ensure that figures meet PLOS requirements. To use PACE, you must first register as a user. Registration is free. Then, login and navigate to the UPLOAD tab, where you will find detailed instructions on how to use the tool. If you encounter any issues or have any questions when using PACE, please email PLOS at [figures@plos.org](mailto:figures@plos.org). Please note that Supporting Information files do not need this step.
